# Self-efficacy centered comprehensive interventions and effects in patients undergoing radical surgery for gastric cancer

**DOI:** 10.3389/fmed.2025.1613280

**Published:** 2025-07-24

**Authors:** Huali Zhou, Rong Bao, Liping Qiu, Yuhan Zhang, Qiong Gu

**Affiliations:** Department of Gastric Surgery, Sichuan Clinical Research Center for Cancer, Sichuan Cancer Hospital & Institute, Sichuan Cancer Center, University of Electronic Science and Technology of China, Chengdu, China

**Keywords:** self-efficacy, comprehensive interventions, gastric cancer, nutritional status, quality of life

## Abstract

**Objective:**

To assess the effects of self-efficacy theory centered comprehensive interventions on perioperative nutritional status, self-management ability, self-efficacy perception, and quality of life in patients undergoing radical surgery for gastric cancer.

**Methods:**

Using a convenience sampling method, 213 patients who underwent radical gastrectomy for gastric cancer at a tertiary specialized hospital in Sichuan Province from October 2023 to April 2024 were selected as the research subjects for randomized controlled trial. The selected patients were randomly assigned to the experimental group with 107 cases and the control group with 106 cases. The experimental group received self-efficacy centered comprehensive interventions including dietary guidance, psychological intervention and counseling, symptom management, exercise guidance, etc. The control group received routine medical interventions. The nutritional status, self-efficacy, self-management ability, and quality of life of the two groups of patients were evaluated at 1 month, 3 months, 6 months, and 1 year after discharge from hospital.

**Results:**

The analytical outcomes across diverse time intervals demonstrated that the nutritional risk of patients in the experimental group was significantly lower than that in the control group, while the self-efficacy scale, self-management ability scale, and quality of life scores in the experimental group were all higher than those in the control group. The differences were statistically significant (*p* < 0.05).

**Conclusion:**

Comprehensive interventions centered on self-efficacy theory can improve the nutritional status, self-efficacy, self-management ability, and quality of life of patients undergoing radical gastrectomy for gastric cancer.

## 1 Introduction

Gastric cancer is a common malignant tumor of the digestive system with the fifth-highest mortality rate among all cancer types globally (1). Early-stage gastric cancer patients often have good prognosis, but the vast majority of patients are already in the stage of local progression when they seek medical treatment (2). About 37% of new gastric cancer cases worldwide come from China (1), and most were already at progressive stage at the time of diagnosis. Standard D2 lymph node radical resection is the preferred treatment option recommended by the guidelines for patients with progressive gastric cancer (3). The physiological state and metabolism of patients change after surgery. Coupled with insufficient nutritional intake and misguided dietary concepts, the incidence of postoperative malnutrition in gastric cancer patients reaches as high as 60% (4). This severely influences the incidence of postoperative complications and the mortality rate, casting a shadow over patients’ recovery and prognosis (5). The heavy disease burden and concerns about the prognosis of the tumor will bring great pressure and psychological stress to patients and their families. Negative emotions such as anxiety and depression directly affect their quality of life (6). After radical gastrectomy for gastric cancer, patients need to go through a long period of self-management. The core of self-management is to improve the ability of patients to manage the disease, which emphasizes the central role of patients (7). Self-efficacy is an important component of self-management (8–10), which not only promotes postoperative recovery but also improves patients’ quality of life and health status. Therefore, this study aims to explore the application of medical interventions centered on self-efficacy theory in patients undergoing advanced gastric cancer radical surgery, and investigate the effect of such comprehensive interventions on improving the nutritional status and quality of life of patients.

## 2 Materials and methods

### 2.1 Patients

This was a randomized controlled trial study. Based on the 8th edition of the Union for International Cancer Control (UICC) clinical TNM staging criteria for gastric cancer, the convenience sampling method was used to select 213 patients who underwent radical surgery for gastric cancer at a tertiary specialized hospital in Sichuan Province from October 2023 to April 2024. The selected patients were randomly divided into an experimental group of 107 cases and a control group of 106 cases. Inclusion criteria: (1) aged between 18 and 75 years old; (2) pathologically diagnosed with gastric malignancy and scheduled for the first elective surgery; (3) underwent R0 radical resection of gastric cancer; (4) without severe organ dysfunction, hypertension, diabetes, etc.; (5) had not received any professional psychological treatment after the disease was diagnosed; (6) obtained informed consent from the patient and their family members; (7) conscious, without cognitive or communication disorders; (8) completed a 1-year follow-up after surgery. Exclusion criteria: (1) severe dysfunction of vital organs, such as liver or kidney failure; (2) with speech disorders like aphasia or deafness who are unable to fill out the questionnaire properly; (3) with mental disorders or unwilling to cooperate; (4) those who withdrew from the survey midway; (5) patients who died in the hospital. This study had been approved by the Ethics Committee of the hospital (Approval No. SCCHEC-02-2023-112), obtained informed consent from patients and their families with signed consent forms. The general information comparison between the two groups of patients is shown in Table 1, and the difference is not statistically significant.

**Table 1:** Comparison of general information between two groups of patients.

Item	Control group (*n* = 106)	Experimental group (*n* = 107)	χ^2^/t	*p*
Age (x ± s, year)	62.75 ± 7.62	60.13 ± 5.97	0.995	0.32
Gender			0.82	0.365
Male	64	71		
Female	42	36		
Cultural level			2.064	0.724
Elementary school and below	15	17		
Junior high school	52	46		
High school or vocational school	29	32		
College and undergraduate programs	9	12		
Master’s degree or above	1	0		
Career			1.196	0.754
Employed	27	31		
Unemployed	32	35		
Retired	45	38		
Student	2	3		
Marital status			2.762	0.43
Married	83	92		
Unmarried	7	4		
Divorced	5	2		
Widowed	11	9		
TNM stage			1.439	0.487
Stage I	32	29		
Stage II	47	56		
Stage III	27	22		
Have dedicated people to take care			1.007	0.316
Yes	95	91		
No	11	16		
Ethnic group			1.391	0.238
Han	87	94		
Minority	19	13		
Surgical method			1.875	0.392
Proximal gastrectomy	27	19		
Distal gastrectomy	64	71		
Total gastrectomy	15	17		
Medical insurance type			1.139	0.768
Self-funded	3	2		
Urban resident medical insurance	47	41		
Urban employee medical insurance	6	7		
New rural cooperative medical Insurance	50	57		
Monthly family income			0.871	0.647
≤3,000 yuan	4	6		
3,000–5,000 yuan	21	17		
≥5,000 yuan	81	84		

### 2.2 Intervention methods

#### 2.2.1 Intervention methods for the control group

During the hospitalization period, patients in the control group routinely completed all screenings, assessments, treatments, and nursing care. When they visited the outpatient department for review or were re-admitted for adjuvant chemotherapy at 1 month, 3 months, 6 months, and 1 year after surgery, they would receive nutritional risk scoring, body composition analysis, self-efficacy assessment, self-management ability assessment, and quality of life scoring. They would also receive examinations of relevant blood-related indicators, and complete relevant follow-up surveys including diet types, food intake, body weight, pain, surgical incision status, catheter color and volume, major discomforts, activity level, medication use, etc. Additionally, health education and relevant consultations would be provided.

#### 2.2.2 Intervention methods for the experimental group

For patients in the experimental group, besides the same screenings, assessments, treatments, and nursing care provided to patients in the control group, the also received the self-efficacy centered comprehensive interventions.

1. Self-Efficacy Centered Interventions

The self-efficacy theory is an important part of psychologist Bandura’s (11) social cognitive theory. Considering the situation of our department, we referred to the Chinese version of the Cancer Self-Management Efficacy Scale (12) and the revised General Self-Efficacy Scale (GSES) (13) and formulated intervention measures after joint deliberation by doctors, nurses, dietitians, rehabilitation therapists, case managers, and psychological counselors. The intervention measures include:

- Dietary guidance: To enhance the nutritional intake, patients without obstruction before surgery should have a balanced and divers diet with various fresh vegetables and fruits, coarse and fine grains, appropriate amount of protein provided by lean meat, fish, egg or milk as well as controlled oil and salt. The daily required energy should be proportionally distributed into three meals. Within 24 to 48 h after the surgery, in accordance with the concept of enhanced recovery after surgery (ERAS), oral intake should be resumed and progress step by step following doctor’s instructions, starting from liquid diet to regular diet (14). After being discharged and returning home, patients should break through dietary prejudices and misunderstandings. They should have a carefully planned diet with regulated daily intake of meat, eggs, milk, vegetables, and fruits, and avoid spicy, coarse or hard foods.
- Psychological intervention and counseling: Patients may have a fear of surgery and worry about post-operative recovery. Physicians should teach basic knowledge about the disease and surgical procedure to patients, while encouraging patients to express their thoughts and improve their compliance. Patients can record the development of their disease and feelings by keeping a diary. If necessary, a multidisciplinary approach with the inclusion of psychological counselors for consultation may help in relieving the psychological pressure of patients.
- Support for reintegration into society: Patients were encouraged to return to normal work and family life. They could participate in social activities regularly, communicate more with family members and friends, release negative and pessimistic emotions, and gradually return to the state before they got sick.
- Assistance on symptom management: Patients who underwent radical gastrectomy may experience symptoms such as: wound pain, sleep disorders, abdominal distension and diarrhea after being discharged from the hospital. Pain can be relieved by various methods such as: meditation, hypnosis, relaxation, diversion, and distraction. Patients with sleep disorders could adjust their daily schedules, create a favorable sleep environment before going to bed, and soak their feet in warm water to improve their sleep. Patients experiencing abdominal distension and diarrhea should avoid greasy and gas-producing foods and chew their food thoroughly. If those efforts fail to relieve the symptoms, patients should seek medical help timely. They should take medications correctly under the guidance of doctors to eliminate the impact of adverse symptoms.
- Prompting proper exercise: According to the concept of ERAS, assistance can be provided for the patient to move in the early postoperative period as soon as the patient can tolerate. The amount of activity can be gradually increased according to the degree of recovery. After being discharged from the hospital, patients should perform moderate intensity exercises three times a week, such as practicing Tai Chi, doing square dancing or jogging. Each exercise session should last for 30 min. Patients could go for walks, listen to music, grow flowers or read books in their spare time.

Upon admission, all patients received relevant screenings and assessments to establish basic files in the case management system. Researchers and patients jointly determined action goals and signed informed consent forms and behavior contracts. Text groups were established on WeChat, a popular messaging app utilized in China, to facilitate communication and cooperations. During the hospitalization, bedside rounds were conducted daily to understand how well the patients understood and applied relevant knowledge. After patients were discharged from the hospital, researchers sent rehabilitation related knowledge to the communication group every week. A follow-up survey was carried out to check their understanding of the given knowledge. Patients who actively participate in answering and showing good compliance were praised. Each month, the patient with the highest score would be selected as the “Learning Star” and posted in the communication group for other patients to learn from. Patients who failed to meet their goals would be encouraged to find out the reasons for poor compliance and failure, which could help them to improve and be successful in the future.

1. Family support

When patients receive their first treatment upon admission, their family members should be informed of the importance of family support throughout the entire treatment period. During hospitalization, researchers should obtain the cooperation and informed consent of family members. When the patient had been discharged after surgery, researchers called the family members monthly to establish a good relationship. Family members were urged to care more about the patient and create a good and warm family atmosphere. They should not only provide attentive care to the patient physically, but also give them strong spiritual support psychologically.

1. Encourage expression

After the patient is admitted, the supervising doctor and nurse should spend more time on communicating with the patient, encouraging them to express their emotions and understanding of the disease, and providing appropriate positive incentives to the patient. After discharge, researchers conducted weekly phone calls with patients and monthly outpatient follow-up face-to-face conversations. Patients who needed to be readmitted to the hospital for adjuvant chemotherapy were asked about their feelings and if they had any additional questions, which were answered during each hospitalization. If they encountered difficulties, they were consoled with motivational words that emphasized their self-efficacy such as “you can definitely do it” and “you can get through this.

1. Cancer prevention, anti-cancer propaganda, peer education

Patients and their families were required to follow the hospital’s official social media account to read the weekly posted contents about cancer prevention and cancer-related knowledge. During the hospitalization period, educational materials on cancer treatment were distributed to patients and their families. Additionally, each hospital ward contained QR codes that directed to a webpage about gastric cancer prevention, treatment, and precautions to take at home. Patients and their families were encouraged to scan the QR codes at any time to further educate themselves. Sharing of experiences between patients was encouraged. A WeChat group labeled “Patient’s Home” was established, in which patients with good compliance and rehabilitation results were invited to share their cancer experiences, medical conditions, and treatment processes - which gave subsequent patients confidence to overcome the disease.

1. Social support

Staff of the community where the patients lived were informed about the recovery status of the patients. Researchers collaborated with community staff to conduct home visits and provide encouragement. Community would help patients with poor economic conditions to seek social assistance as well.

### 2.3 Research tool

#### 2.3.1 Nutritional risk screening 2002

NRS2002 was introduced in 2003 for screening and evaluating individuals’ nutritional risk status (15). The total score is calculated by add three scores: nutritional status score (0–3 points), disease severity score (0–3 points) and age score (1 point if age is larger than 70 years). The total score ranges from 0 to 7 points. The patient is determined to have nutritional risk and need further malnutrition assessment if the NRS2002 score is equal or larger than 3 points. The Cronbach’s α coefficient of this scale is 0.760, and the criterion-related validity is 0.670.

#### 2.3.2 Patient-generated subjective global assessment

PG-SGA was proposed by Ottery (16) from the United States in 1994, specifically designed for assessing malnutrition in cancer patients (17). PG-SGA would be evaluated if the NRS2002 score is equal or larger than 3. The first part of PG-SGA evaluates weight, food intake, symptoms, activity and physical function that are performed by the patient. The total score is denoted as score A. The second part would be performed by the doctor and includes disease related nutritional needs, metabolic needs and physical examination. The results are denoted as score B, C, D, respectively. The sum of A, B, C, D is the total score which ranges from 0 to 35. Higher scores indicate poorer nutritional status. The quantitative evaluation score is converted into qualitative assessment result by dividing the nutritional status into three levels: well nourished (0–1 points), suspected or moderate malnutrition (2–8 points), severe malnutrition (8–35 points).

#### 2.3.3 Strategies used by people to promote health

SUPPH is a measure of self-reported self-efficacy (18). The measure consists of 28 items that belongs to three aspects: positive attitude, self-determination, and self-stress reduction. A 5-point scoring system (1–5 points) is used, results in a total score of 28–140 points. The higher the score, the higher the patient’s self-management efficacy (11). Patients with SUPPH higher than 65 are determined to have good self-management efficacy, otherwise poor self-management efficacy is determined. The Cronbach’s alpha coefficient of this scale is 0.97.

#### 2.3.4 Self-management ability scale

The self-developed self-management ability scale was used to evaluate the self-management ability of two groups of patients before and after intervention. The scale includes four dimensions: scientific diet, reasonable exercise, following medical advice to take medication, and regular reexamine. Each dimension has a maximum score of 20 points. Higher score indicates better the self-management ability.

#### 2.3.5 The MOS item short from health survey

This survey contains 8 aspects of content, which can evaluate the patient’s physiological function, physiological function, mental health, vitality, emotional function, social function, physical pain perception, and overall health (19). The total score is 100 points. Higher score indicates better their quality of life.

### 2.4 Data collection

The patients completed relevant screening and evaluation after admission. Then, they are informed the purpose and significance of this study. After obtaining the informed consent of the patient and their family members and having them sign the informed consent form, record files were established in the management system. The patients would receive surveys on NRS2002, PG-SGA, SUPPH, SMAS, SF-36 when they performing outpatient review or being readmitted to the hospital. All questionnaires will be distributed and collected on-site. The data were organized and verified by double checking to ensure the accuracy of the data.

### 2.5 Statistical analysis

The SPSS 21.0 was used to perform statistical analysis of the data. Counting data were expressed as numbers and percentages, while metric data were expressed as “mean ± standard deviation.” The comparison of means between the two groups was conducted using Student’ *t*-test. The comparison of means at different observation time points between groups was conducted using two factor repeated measures analysis of variance. The comparison of inter group rates was conducted using the $\chi^{2}$ test. *p* < 0.05 indicates statistical significance of the difference.

## 3 Results

### 3.1 General information comparison

There were 217 patients involved in this study. Two patients in the control group withdrew from this study due to changes in their condition, and two patients in the experimental group withdrew from this study due to personal and family reasons. 213 patients underwent radical gastrectomy for gastric cancer were included, including 107 patients in the experimental group and 106 patients in the control group. The general information comparison between the two groups of patients is shown in Table 1, and there is no statistically significant difference (*p* > 0.05).

### 3.2 Nutritional risk occurrence comparison at different times after surgery

The incidence of nutritional risk (NRS 2002 ≥ 3) in the experimental group was always lower compared to the control group at 1 month, 3 months, 6 months, and 1 year after surgery. The incidence of nutritional risk in the control group was 69.81% at 6 months after surgery, almost the same as the incidence rate 66.99% at 1 month after surgery. In contrast, the incidence of nutritional risk in the experimental group was 49.53% at 6 months after surgery, a decrease of 9.35% from the value of 58.88% at 1 month after surgery. After 1 years of intervention, the nutritional risk in the experimental group was further reduced to 37.74%, lower than the value of 49.06% in the control group with a large margin. The comparison of nutritional risk between two groups of patients at 1 month, 3 months, 6 months, and 1 year after surgery showed statistically significant differences (*p* < 0.05), as shown in Table 2.

**Table 2:** Comparison of nutritional risk (NRS2002 ≥ 3) occurrence between two groups of patients at different time points [case (%)].

Time	Control group (*n* = 106)	Experimental group (*n* = 107)	Time effect F-value	*p*	Inter-group *F*-value	*p*	Time-group interaction *F*-value	*p*
			5.572	0.031	4.118	0.029	9.274	0.015
1 Month	71 (66.99)	63 (58.88)						
3 Month	63 (59.43)	54 (50.47)						
6 Month	74 (69.81)	53 (49.53)						
12 Month	52 (49.06)	41 (37.74)						

### 3.3 Malnutrition incidence comparison at different times after surgery

The proportions of patients with suspected or moderate malnutrition (2 ≤ PG-SGA ≤ 8) and severe malnutrition (PG-SGA ≥ 9) in the experimental group were both lower than that of the control group. After observing for 1 year, the proportion of suspected or moderate malnutrition in the control group decreased by 18.86%, while the experimental group decreased by 21.5%. The percentages of severe malnutrition cases in both of the control group and the experimental group experienced no significant changes during the study. However, the incidences of severe malnutrition in the experimental group were all lower than the incidences in the control group, with gaps between 3.86–8.54%. The statistical comparison of incidences of malnutrition between two groups of patients at 1 month, 3 months, 6 months, and 1 year after surgery was statistically significant (*p* > 0.05), as shown in Table 3.

**Table 3:** Comparison of the incidence of malnutrition (PG-SGA ≥ 2 points) between two groups of patients at different time points [Example (%)].

Item	Time	Control group (*n* = 106)	Experimental group (*n* = 107)	Time effect F-value	*p*	Inter-group F-value	*p*	Time-group interaction *F*-value	*p*
PG-SGA (2–8)				3.125	0.038	4.118	0.022	7.763	0.031
	1 Month	57 (53.77)	58 (54.21)						
	3 Month	49 (46.23)	44 (41.12)						
	6 Month	63 (59.43)	47 (43.93)						
	12 Month	37 (34.91)	35 (32.71)						
PG-SGA ≥ 9				2.236	0.017	3.053	0.043	4.562	0.047
	1 Month	14 (13.21)	5 (4.67)						
	3 Month	14 (13.21)	10 (9.35)						
	6 Month	11 (10.38)	6 (5.61)						
	12 Month	15 (14.15)	6 (5.61)						

### 3.4 Self-management efficacy scale score comparison at different times after surgery

The comparison of SUPPH scores between two groups of patients in Table 4 showed statistically significant difference in terms of time, inter group, and time-group interaction (group *F* value = 2.175, time F value = 3.698, time-group interaction F value = 6.532, *p* < 0.05). The SUPPH scores in both groups consistently improved over time. Nevertheless, the difference of SUPPH scores between two groups gradually increased. As demonstrated in Table 4 the gap between the mean SUPPH score of two groups increased from 1.43 at 1 month after surgery to 5.01 at 1 year after surgery.

**Table 4:** Comparison of scores on the self-management efficacy scale between two groups of patients at different time points (points, $\bar{x\;}\pm s$ ).

Groups	1 Month	3 Month	6 Month	12 Month	Time effect F-value	Inter-group F-value	Time-group interaction F-value
					3.698	2.175	6.532
Control group (*n* = 106)	50.15 ± 7.72	50.34 ± 8.06	51.52 ± 7.78	52.63 ± 6.64			
Experimental group (*n* = 107)	51.58 ± 4.61	53.72 ± 6.42	56.16 ± 7.13	57.64 ± 7.67			
*t*	4.578	2.069	7.442	10.539			
*p*	0.038	<0.001	<0.001	<0.001	0.003	0.012	0.032

### 3.5 Self-management ability scores comparison at different times after surgery

Patients in the experimental group had higher SMAS scores than patients in the control group in terms of all measured dimensions, including scientific diet, reasonable exercise, medication adherence, and regular re- examination, and the difference was statistically significant (*p* < 0.05), as shown in Table 5. For patients in the experimental group, after receiving 1 year of comprehensive interventions, the mean score of scientific diet increased from 11.67 to 18.5, the mean score of reasonable exercise increased from 12.52 to 19.25, the mean score of medication adherence increased from 13.54 to 19.43, and the mean score of regular re-examinations increased from 13.57 to 19.26. The increasing in mean scores in all measured dimensions demonstrated the benefit of receiving the comprehensive intervention. On the contrary, the increasing in the mean scores in various aspects for patients in the control group was significant less.

**Table 5:** Comparison of self-management ability scores between two groups of patients at different time points (points, $\bar{x\;}\pm s$ ).

Item	Time	Control group (*n* = 106)	Experimental group (*n* = 107)	Time effect F-value	*p*	Inter-group F-value	*p*	Time-group interaction *F*-value	*p*
Scientific diet				1.058	0.012	2.373	0.035	4.167	0.002
	1 Month	10.23 ± 1.62	11.67 ± 2.12						
	3 Month	11.18 ± 2.66	13.52 ± 3.17						
	6 Month	12.43 ± 2.72	15.92 ± 3.78						
	12 Month	14.57 ± 3.03	18.5 ± 2.03						
Reasonable exercise				1.068	0.032	3.672	0.047	4.609	<0.001
	1 Month	11.17 ± 1.48	12.52 ± 2.13						
	3 Month	12.90 ± 2.33	14.43 ± 1.67						
	6 Month	13.52 ± 2.67	17.74 ± 2.92						
	12 Month	14.64 ± 2.17	19.25 ± 1.78						
Medication adherence				2.346	0.039	4.058	<0.001	7.679	<0.001
	1 Month	12.52 ± 2.13	13.54 ± 2.52						
	3 Month	13.17 ± 2.64	15.58 ± 1.78						
	6 Month	14.53 ± 2.11	16.76 ± 1.57						
	12 Month	15.63 ± 3.03	19.43 ± 0/68						
Regular re-examination				1.175	0.017	3.673	<0.001	5.772	<0.001
	1 Month	10.33 ± 2.43	13.57 ± 2.12						
	3 Month	11.50 ± 2.17	14.79 ± 1.67						
	6 Month	12.15 ± 1.72	16.34 ± 1.74						
	12 Month	13.36 ± 1.08	19.26 ± 0.78						

### 3.6 Quality of life scores comparison at different times after surgery

The SF-36 score of the experimental group patients was significantly higher than that of the control group, and the difference was statistically significant (*p* < 0.05), as shown in Table 6. The positive impact of the self-efficiency centered comprehensive interventions on the quality of life for gastric patients after surgery is obvious verified by the increasing in all evaluated dimensions.

**Table 6:** Comparison of quality-of-life scores between two groups of patients at different time points (points, $\bar{x\;}\pm s$ ).

Item	Time	Control group (*n* = 106)	Experimental group (*n* = 107)	Time effect F-value	*p*	Inter-group F-value	*p*	Time-group interaction F-value	*p*
Physiological function				1.073	0.032	12.063	0.002	5.075	<0.001
	1 Month	65.52 ± 4.58	64.47 ± 4.67						
	3 Month	67.32 ± 3.65	67.73 ± 4.33						
	6 Month	68.74 ± 4.13	70.49 ± 3.57						
	12 Month	70.63 ± 3.63	73.28 ± 3.12						
Physiological functions				2.352	<0.001	13.069	<0.001	23.672	<0.001
	1 Month	61.52 ± 4.17	63.25 ± 3.16						
	3 Month	62.74 ± 3.69	66.53 ± 4.23						
	6 Month	65.57 ± 4.33	69.17 ± 4.54						
	12 Month	69.26 ± 3.18	72.27 ± 3.47						
Physical pain				7.073	0.015	2.562	0.037	13.574	0.003
	1 Month	65.54 ± 4.31	67.09 ± 4.03						
	3 Month	67.09 ± 3.78	69.73 ± 4.23						
	6 Month	67.47 ± 4.02	71.25 ± 3.56						
	12 Month	69.33 ± 3.45	73.57 ± 3.18						
General health condition				6.706	<0.001	3.175	<0.001	25.76	<0.001
	1 Month	68.23 ± 5.13	67.53 ± 4.58						
	3 Month	69.72 ± 4.71	69.18 ± 3.76						
	6 Month	70.43 ± 5.57	73.43 ± 4.03						
	12 Month	71.26 ± 4.18	75.79 ± 3.43						
Energy				11.574	0.028	7.653	<0.001	23.689	<0.001
	1 Month	68.56 ± 4.68	69.23 ± 4.92						
	3 Month	68.52 ± 4.73	72.75 ± 5.03						
	6 Month	70.18 ± 5.06	74.76 ± 5.42						
	12 Month	72.63 ± 4.16	77.29 ± 5.17						
Social function				7.726	<0.001	9.058	<0.001	20.125	<0.001
	1 Month	73.18 ± 4.76	74.26 ± 4.43						
	3 Month	75.63 ± 5.18	76.57 ± 4.78						
	6 Month	76.74 ± 4.69	79.43 ± 5.16						
	12 Month	77.52 ± 5.13	83.49 ± 4.71						
Emotional function				12.736	<0.001	17.668	0.013	22.373	<0.001
	1 Month	72.15 ± 5.63	73.25 ± 5.15						
	3 Month	73.47 ± 5.22	75.67 ± 5.42						
	6 Month	74.76 ± 4.89	78.46 ± 4.72						
	12 Month	76.53 ± 4.48	81.15 ± 4.43						
Mental health				9.053	0.002	6.235	<0.001	17.592	0.003
	1 Month	70.52 ± 4.77	69.73 ± 4.23						
	3 Month	71.43 ± 5.13	71.05 ± 5.52						
	6 Month	72.75 ± 4.32	73.46 ± 4.83						
	12 Month	74.06 ± 5.58	75.79 ± 4.34						
Total score				6.097	0.023	13.508	<0.001	15.069	0.002
	1 Month	69.26 ± 4.78	69.57 ± 5.12						
	3 Month	70.33 ± 4.63	71.47 ± 4.77						
	6 Month	71.79 ± 5.22	74.26 ± 5.28						
	12 Month	73.67 ± 4.73	78.49 ± 5.73						

## 4 Discussion

Self-efficacy is a critical psychological resource for cancer patients. This study developed a self-efficacy centered comprehensive intervention program for patients with gastric cancer. Results shown that such interventions could effectively enhance self-efficacy in gastric cancer patients, which helped to reduce the incidence of malnutrition, enhance the self-management ability and improve the quality of life. These findings are consistent with previous studies (9, 10, 20–22), demonstrating the effectiveness of the proposed comprehensive intervention.

### 4.1 Self-efficacy centered comprehensive interventions can reduce the nutritional risk and incidence of malnutrition in patients undergoing radical gastrectomy

Research has shown that malnutrition is a substantial problem for patients undergoing radical gastrectomy for gastric cancer (23), which can persist until 12 months after surgery before gradually recovering. The malnutrition status of gastric cancer patients after surgery may increase the risk of patient death and cancer recurrence (24–26). Therefore, it is necessary to pay attention to the long-term nutritional status of patients after radical gastrectomy for gastric cancer. In clinical practice, many indicators related to nutrition are used for nutritional evaluation, such as BMI, grip strength, subcutaneous fat thickness, and albumin (27, 28). However, such indicators are not suitable or convenient for evaluating the nutritional status of postoperative home patients. Therefore, this study used NRS2002 screening to assess individual nutritional risk status, and used PG-SGA specifically designed for cancer patients for malnutrition assessment. After receiving self-efficacy centered comprehensive intervention, the experimental group had significantly fewer patients with NRS2002 ≥3 and PG-SGA ≥2 at 1 month, 3 months, 6 months, and 1 year after discharge compared to the control group, and the difference was statistically significant (*p* < 0.05). Moreover, the number of patients with severe malnutrition evaluated by PG-SGA was significantly lower than that of the control group, which was consistent with other research results (29). Therefore, self-efficacy centered comprehensive interventions can reduce the nutritional risk and incidence of malnutrition in patients undergoing radical gastrectomy for gastric cancer. In this study, psychological intervention and counseling were provided to patients upon admission, with the premise of encouraging positive attitudes. Through peer education and online and offline learning conducted by the department, patients’ understanding of the disease was further improved, so that patients and their families could truly realize the importance of nutrition management. During the treatment period, researchers also specifically answered patients’ diet related questions and required active support from patients’ families, which helped to implement patients’ dietary plans and greatly improve their nutritional status.

### 4.2 Self-efficacy centered comprehensive interventions improved the self-efficacy of patients after radical gastrectomy for gastric cancer

Patients in the experimental group received self-efficacy centered comprehensive interventions, which attributed to the higher self-management efficacy scores than the control group at 1 month, 3 month, 6 months, and 1 year after discharge (*p* < 0.05), indicating that medical interventions based on self-efficacy theory can help improve the self-efficacy of postoperative gastric cancer patients. In this study, researchers proposed health interventions based on patients’ personal beliefs, and multidimensional measures were taken to enhance patients’ self-efficacy. Firstly, the role of nurses was strengthened in the comprehensive intervention program, allowing them to closely monitor the status of patients and help patients to form a firm belief in rehabilitation after discharge. Secondly, family and society members were fully motivated to provide patients with emotional and practical support. Moreover, through the positive guidance of peer education, patients’ compliance was enhanced and therefore resulted in improved self-care capabilities compared to the control group. These measures provided psychological support to patients from different aspects, thus effectively enhancing their self-efficacy, which is consistent with previous studies (20, 30). Last but not least, through the use of a mini-program on WeChat, online learning classes regularly held by medical professional (nutritionists, doctors and nurses), and distribution of additional educational materials through a QR code, caring services can be continuously carried out to strengthen both patient education and the doctor-patient relationship, further enhancing patients’ self-efficacy.

### 4.3 Self-efficacy centered comprehensive interventions improved the self-management ability of patients after radical gastrectomy for gastric cancer

Higher scores in self-management ability (scientific diet, reasonable exercise, following medical advice, regular follow-up) during all study period for patients in the experimental group indicated that self-efficiency centered comprehensive interventions could fully stimulate the subjective initiative of gastric cancer patients. This allowed them to actively participate in self-management, rather than relying solely on family members or caregivers. Patients became decision makers of self-healing measures. Research has shown that the improvement of self-management ability is helpful for post-operative recovery (31). Moreover, guidance on dietary, exercise, and symptom management, along with support from family and the community, laid the foundation for patients to independently carry out daily life activities in the future.

### 4.4 Self-efficacy centered comprehensive interventions was beneficial for improving patients’ quality of life

Studies have shown that patients undergoing chemotherapy after radical gastrectomy for gastric cancer may experience varying degrees of adverse reactions such as: nausea, vomiting, decreased appetite, and fatigue, which can affect their mood, sleep, and energy levels, leading to declined physical function (32). Limited physical function and restricted mobility would decrease patients’ confidence and self-efficacy (33). Moreover, cancer treatments and chemotherapy drugs might induce cognitive impairment, which negatively impact patients’ memory, attention, and processing speed (34). All those factors lead to an overall decrease in patient quality of life (35). In this study, patients in the experimental group patients had higher scores in all dimensions of the SF-36 scale at various time intervals after discharge than patients in the control group (*p* < 0.05). This indicated that quality of life is closely related to the adopted intervention measures. The proposed self-efficiency centered comprehensive interventions can improve patients’ understanding of the disease, alleviate their negative emotions, provide a favorable theoretical basis for their recovery, and eventually improve their quality of life.

## 5 Conclusion

This study aimed to explore the effectiveness of self-efficacy centered comprehensive intervention measures for patients after radical gastrectomy for gastric cancer, with the hope to establish a management model for the entire rehabilitation period of gastric cancer patients which can improve their nutritional status, self-management ability, self-efficacy, and quality of life. Before the surgery, an MTD team were gathered including the chief physician, chief nurse, pathologist, radiologist, nutritionist, anesthesiologist, and case manager to provide comprehensive guidance and intervention to the patient. After the surgery, the chief physician, chief nurse, nutritionist, rehabilitation specialist, and case manager also provided a series of intervention measures and extended the service outside the hospital. During this process, we fully mobilized and exerted the patient’s subjective initiative, made them realize the value of self-efficacy, and carried out self-efficacy psychotherapy as much as possible to improve the nutritional and psychological status of gastric cancer patients after radical surgery. Currently the number of patients included in this study is limited, and the research time is relatively short. In addition, current study only focuses on the impact of the proposed comprehensive intervention measures on patients’ self-efficacy, nutritional status and quality of life. In the future, we hope to have more enrolled patients to participate the study, and machine learning techniques will be used to conduct more in-depth analysis of the data to study the impact of improved patient self-efficacy on patient impact risks and quality of life. It is hoped to find more scientific and reasonable comprehensive interventions plans for gastric cancer patients in practice, and provide useful tools for the implementation of such comprehensive interventions.

## Data Availability

The raw data supporting the conclusions of this article will be made available by the authors, without undue reservation.
